# Study on QSTR of Benzoic Acid Compounds with MCI

**DOI:** 10.3390/ijms11041228

**Published:** 2010-03-24

**Authors:** Zuojing Li, Yezhi Sun, Xinli Yan, Fanhao Meng

**Affiliations:** 1 School of Foundation, Shenyang Pharmaceutical University, No. 103 Wenhua Road, Shenyang, Liaoning, 110016, China; E-Mail: zuojing100677@sina.com (Z.L.); yanlier@hotmail.com (X.Y.); 2 School of Pharmaceutical Science, China Medical University, No. 92 Bei-er Road, Shenyang, Liaoning, 110001, China; E-Mail: sunyezhi@gmail.com (Y.S.)

**Keywords:** benzoic acid, acute toxicity, MCI, toxicity prediction, QSTR

## Abstract

Quantitative structure-toxicity relationship (QSTR) plays an important role in toxicity prediction. With the modified method, the quantum chemistry parameters of 57 benzoic acid compounds were calculated with modified molecular connectivity index (MCI) using Visual Basic Program Software, and the QSTR of benzoic acid compounds in mice *via* oral LD_50_ (acute toxicity) was studied. A model was built to more accurately predict the toxicity of benzoic acid compounds in mice *via* oral LD_50_: 39 benzoic acid compounds were used as a training dataset for building the regression model and 18 others as a forecasting dataset to test the prediction ability of the model using SAS 9.0 Program Software. The model is LogLD_50_ = 1.2399 × ^0^J^A^ +2.6911 × ^1^J^A^ – 0.4445 × J^B^ (R^2^ = 0.9860), where ^0^J^A^ is zero order connectivity index, ^1^J^A^ is the first order connectivity index and J_B_ = ^0^J^A^ × ^1^J^A^ is the cross factor. The model was shown to have a good forecasting ability.

## 1. Introduction

**Figure 1.**
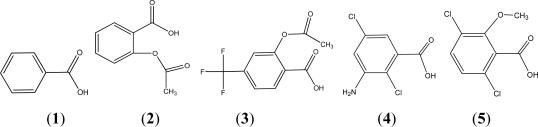
Molecular structures of benzoic acid (**1**), aspirin (**2**), triflusal (**3**), chloramben (**4**) and dicamba (**5**).

Benzoic acid compounds are an important organic chemical raw material that are widely used in food, medicine, cosmetic, antiseptic, insecticide, dyestuff, *etc.* For example, benzoic acid is a common antiseptic, Aspirin is a famous non-steroid anti-inflammatory drug, Triflusal is a antithrombotic, and Chloramben and Dicamba are common pesticides (see Figure 1). Most benzoic acid compounds are toxic and are hardly degraded by microorganism in the natural environment, which may cause serious public health and environmental problems.

With the development of synthetic chemistry, combinatorial chemistry and pharmaceutical chemistry, millions of new compounds are being synthesized. Classical chemical substance evaluation needs a lot of time and is expensive, and the speed of analyzing the toxicity of compounds is less than the speed of discovery of new compounds. Nowadays, scientists pay more and more attention to the importance of prediction toxicity in the early stage. Quantitative structure-toxicity relationships (QSTR) have been efficiently used for the study of toxicity mechanisms of various compounds [1].

QSTR plays an important role in toxicity forecasting, which is widely used in the modern studying of compounds, since more and more compounds are being found. It is necessary to predict the toxicity of compounds accurately and quickly [2–4]. QSTR of benzoic acid compounds with molecular connectivity index (MCI) in mice *via* oral LD_50_ (acute toxicity, half lethal dose) are not reported. The quantitative structure characteristic parameters of 57 benzoic acid compounds were obtained with MCI. Values of LD_50_ for mice in benzoic acid compounds have been collected from various literature sources. In this work, the QSTR of benzoic acid compounds in mice *via* oral LD_50_ was studied and a model was developed to more accurately predict the toxicity of benzoic acid compounds in mice *via* oral LD_50_. 39 benzoic acid compounds were used as a training dataset for building the regression model, and 18 other benzoic acid compounds as a forecasting dataset to test the prediction ability of the model. The experimental result analysis showed that ^0^J^A^, ^1^J^A^ and cross factor J^B^ were important factors affecting the toxicity of benzoic acid compounds (although the toxicity mechanism of compounds is not clear yet), where ^0^J^A^ is zero order connectivity index, ^1^J^A^ is the first order connectivity index and J_B_= ^0^J^A^ × ^1^J^A^ is the cross factor.

## 2. Research Methods

In 1975, Milan Randic described a skeletal branching index that correlated with the three physical properties of alkenes [5]. The concept was further developed and applied extensively by Kier and Hall [6–8], which led to the molecular connectivity index (MCI). Eventually, Kier and Hall modified the connectivity indices to discriminate carbon atoms from other heteroatoms, which introduced the valance molecular connectivity index ^m^χ^t^ [9]. The MCI is calculated with the follow formula:

(1) $${}^{\text{m}}\chi^{\text{t}}=\sum {{}^{\text{Nm}}}_{\text{j}=1}{\left(\Pi^{\text{m}+1}{}_{\text{i}}\;1/\delta_{\text{i}}\right)}^{1/2}$$

^m^χ^t^ is mth-order MCI, *t* is the type of sub-graph including path (p), cluster (c), path-cluster (pc), N_m_ is the number of the sub-graph of the same type and order. The abbreviation is δ = σ – *h*, where σ is the count of electrons in σ orbital and *h* is the count of bonding hydrogen atoms.

There was no doubt that the MCI was proved to be the one of the most successful and widely used descriptors. The MCI has been introduced and used in many studies [10–13].

From the skeletal branching index of Randic to the connectivity index modified by Kier and Hall, the core is the connectivity of atoms, which is from the connectivity δ_i_ of upper atom to valence connectivity of δ_iv_. The computing method of heteroatom i modified by Kier and Hall is as the following formula:

(2) $$\delta_{\text{iv}}=\left({\text{Z}}_{\text{i}}-{\text{h}}_{\text{i}}\right)/\left(\text{Z}-{\text{Z}}_{\text{i}}-1\right)$$

Z and Z_i_ are the count of extra nuclear electrons and valence electrons, respectively, *h*_i_ is the count of hydrogen atoms combining with heteroatom i. Although Kier *et al* contributed to the computing method of heteroatom i, the method could not discriminate the same heteroatom in different oxidation states. More recently, Yu *et al* improved the method, and redefined the valence connectivity value δ^h^_i_ using the following formula [14]:

(3) $$\delta^{\text{h}}{}_{\text{i}}=2\;\times \;\text{Z }\left({\text{Z}}_{\text{i}}-{\text{h}}_{\text{i}}\right)\left[{\left(8-{\text{N}}_{\text{i}}\right)}^{1/{\text{N}}_{\text{i}}}\right]\left[{\left(2{\text{n}}_{\text{i}}-1\right)}^{{\text{h}}_{\text{i}}/{\text{N}}_{\text{i}}-1}\right]/\left[\left({\text{m}}_{\text{i}}+{\text{L}}_{\text{p}}\right)\left(2{\text{n}}_{\text{i}}-1\right)\right]$$

m_i_ is the count of bonding electrons, Z is the count of extra nuclear electrons, n_i_ is maximum first quantum number, Z_i_ is the valence electron number, N_i_ is the count, L_p_ is the hybridization style of heteroatom i, the value as following: sp^3^, L_p_ = 1; sp^2^, L_p_ = −1.8; sp, L_p_ = 2; if that is the atom itself, L_p_ = 2, m_i_ = 0.

The program package for calculating the MCI of compounds was compiled by Visual Basic Program Software according to the modified formula. In order to predict the toxicity of benzoic acid compounds and get the prediction model, the molecular structure of 57 benzoic acid compounds was entered into the program package and their MCI were calculated. 39 of them were a training dataset for building the multi variance linear regression model (logarithm of LD_50_ as dependent variable and MCI as factor), and 18 of them were predicted samples to test the prediction ability of the model using SAS 9.0 Program Software. During the process of building the regression model, the cross factor was considered into the model.

## 3. Results and Discussion

In what follows, we will present the process of computing MCI, choosing factors of the regression model and building the model, as well as testing the model. Firstly, zero order connectivity index ^0^J^A^ and first order connectivity index ^1^J^A^ were calculated using the program package. The value of LD_50_ was converted to logarithm in order to make all the data in the same order of magnitude and easier to statistically analysze and compare. Then, the toxicity data was analyzed in the training dataset as regression analysis. Non-intercept stepwise regression was chosen as the statistical method. The influencing factors were as follows: zero order connectivity index ^0^J^A^, first order connectivity index ^1^J^A^ and the cross factor J_B_= ^0^J^A^ × ^1^J^A^. These influencing factors were inspected, and the results were as below:

1. ^0^J^A^: R-Square = 0.9542 and C_(p)_ = 1.0000
2. ^1^J^A^: R-Square = 0.9560 and C_(p)_ = 1.0000
3. J^B^: R-Square = 0.8656 and C_(p)_ = 1.0000
4. ^0^J^A^, ^1^J^A^: R-Square = 0.9560 and C_(p)_ = 0.2565
5. ^0^J^A^, J^B^: R-Square = 0.9829 and C_(p)_ = 2.0000
6. ^1^J^A^, J^B^: R-Square = 0.9816 and C_(p)_ = 2.0000
7. ^0^J^A^, ^1^J^A^: J^B^: R-Square = 0.9860 and C_(p)_ = 3.0000

The results show that the groups are fine expect (3) and (4), and correlation coefficient (R^2^) showed that (7) is the best. It was shown that the regression linearity of (7) is better than other groups. Therefore, ^0^J^A^, ^1^J^A^ and J^B^ were chosen as the independent variables of the model (see Table 1).

Comparing the p value in the table, it was shown that ^0^J^A^, ^1^J^A^ and J^B^ had an obvious significant influence, and a regression estimated model was built:

$${\text{LogLD}}_{50}=1.2399\;\times {\;}^{0}{\text{J}}^{\text{A}}\;+\;2.6911\;\times {\;}^{1}{\text{J}}^{\text{A}}-0.4445\;\times \;{\text{J}}^{\text{B}}\;\left({\text{R}}^{2}=0.9860\right)$$

**Figure 2.**
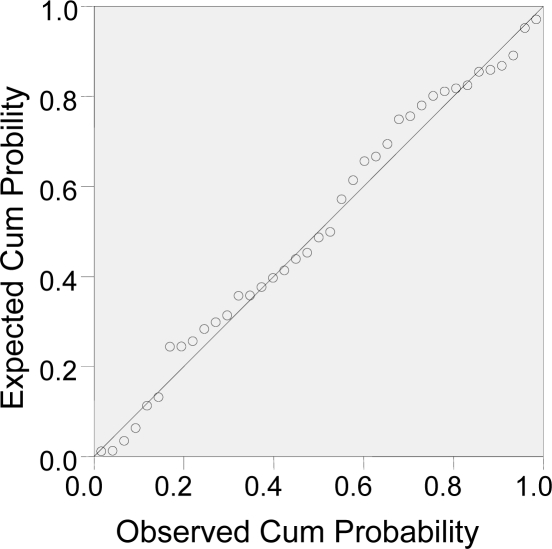
Normal P-P Plot of residual.

Obeying the principles that the value of correlation coefficient (R^2^) is approximate to 1 and the p value is less than 0.01, as well as the numbers of the parameters equal to the test coefficient, we found that the linearity of the model is appropriate. The result of residual analysis shows that the fitting of the model was good (see Table 2). The distribution of residual is a normal distribution, since the scatter plots are almost standing on one line (see Figure 2).

From analysis of the model, it was known that ^0^J^A^, ^1^J^A^ and cross factor J^B^ had great influence on the oral toxicity in mice. When ^0^J^A^ and ^1^J^A^ decrease, the value of LD_50_ increases. And LD_50_ decreases as J^B^ increases. Since increasing LD_50_ resulted in lower toxicity, therefore, the model showed that ^0^J^A^ and ^1^J^A^ have a negative correlation to the toxicity of benzoic acid compounds, and J^B^ has a positive correlation to the toxicity of benzoic acid compounds. The ability of regression model with 18 benzoic acid compounds was also tested, and the result indicates that the prediction ability of the model is good (Table 3). It is shown that these influencing factors indeed had an significant effect on toxicity, and the forecasting accuracy of the model becomes higher when introducing the cross factor (J^B^).

## 4. Conclusions

LD_50_ is a common factor for evaluating compound toxicity, which reflects receptivity of test animals, and LD_50_ values have high reproducibility and stability. In QSTR study, linear regression analysis is a widely useful quantization method [15]. In this work, the quantitative parameters were calculated with MCI and the toxicity prediction model of benzoic acid compounds was obtained as follow. LogLD_50_=1.2399 × ^0^J^A^ +2.6911 × ^1^J^A^ – 0.4445 × J^B^, R-Square = 0.9860. The model has a good forecasting ability.

## Figures and Tables

**Table 1.** Variable parameter estimation analysis.

**Variable parameters**	**Standard estimate**	**Error**	**Type II SS**	**F value**	**Pr > F**
^0^J^A^	1.2399	0.4374	6.6827	8.04	0.0075
^1^J^A^	2.6911	0.8057	9.2768	11.16	0.0020
J^B^	–0.4445	0.0509	63.3327	76.16	<0.0001

**Table 2.** Building the toxicity prediction regression model of benzoic acid compounds with training dataset (39 benzoic acid compounds).

**No.**	**Compound**	**CAS No.**	**LogLD_50_**		**Std error (predicted)**	**Residual**

			**Dependent variable**	**Predicted value**		
1	benzamide	55-21-0	7.056	7.187	0.189	–0.131
2	4-aminobenzoic acid	150-13-0	7.955	7.264	0.174	0.691
3	4-chlorobenzoic acid	74-11-3	7.065	7.254	0.175	–0.189
4	3-hydroxybenzoic acid	99-06-9	7.601	7.236	0.176	0.362
5	4-bromobenzoic acid	586-76-5	6.965	7.283	0.172	–0.318
6	2-iodobenzoic acid	88-67-5	7.313	7.310	0.170	0.003
7	amino salicylic acid	65-49-6	8.294	7.334	0.169	0.960
8	methyl benzoate	93-58-3	8.111	7.490	0.172	0.621
9	3-aminobenzoic acid	99-05-8	8.748	7.264	0.174	1.484
10	3-methylbenzoic acid	99-04-7	7.396	7.496	0.151	–0.100
11	4-hydroxybenzoic acid	99-96-7	7.696	7.239	0.176	0.457
12	4-methylbenzoic acid	99-94-5	7.758	7.496	0.151	0.262
13	6-methylsalicylic acid	567-61-3	5.522	7.518	0.155	–1.997
14	3,5-diiodosalicylic acid	133-91-5	6.109	7.460	0.170	–1.351
15	2-acetyloxybenzoic acid (aspirin)	50-78-2	5.522	7.493	0.201	–1.971
16	2,4,6-triiodobenzoic acid	2012-31-9	7.170	7.490	0.170	–0.320
17	3,4,5-triiodobenzoic acid	2338-20-7	8.434	7.490	0.170	0.944
18	4-tert-butylbenzoic acid	98-73-7	6.342	6.617	0.481	–0.274
19	2-formylbenzoic acid	119-67-5	8.407	7.411	0.160	0.997
20	2-hydroxybenzamide (salicylamide)	65-45-2	5.704	7.306	0.181	–1.603
21	2-hydroxybenzoic acid (salicylic acid)	69-72-7	6.174	7.243	0.176	–1.069
22	2-aminobenzoic acid methyl ester	134-20-3	8.269	7.513	0.196	0.756
23	2-(acetyl amino)benzoic acid	89-52-1	7.016	7.481	0.405	–0.465
24	2-amino-3,5-dichlorobenzoic acid	2789-92-6	7.185	7.412	0.173	–0.227
25	4-hydroxy-3,5-diiodobenzoic acid	618-76-8	8.294	7.460	0.170	0.834
26	3,5-diiodo-4-methoxybenzoic acid	4253-11-6	6.908	7.484	0.280	–0.576
27	2,3,6-trichlorobenzoic acid (2,3,6-TBA)	50-31-7	6.422	7.408	0.172	–0.986
28	2-aminobenzoic acid (anthranilic acid)	118-92-3	7.244	7.268	0.174	–0.024
29	4-aminobenzoic acid ethyl ester (benzocaine)	94-09-7	7.824	7.437	0.235	0.387
30	2-hydroxybenzoic acid methyl ester	119-36-8	7.012	7.516	0.747	–0.504
31	2,5-dihydroxybenzoic acid (gentisic acid)	490-79-9	8.412	7.313	0.171	1.099
32	5-amino-2-hydroxybenzoic acid (mesalamine)	89-57-6	8.123	7.334	0.169	0.789
33	3-amino-2,5-dichlorobenzoic acid (chloramben)	133-90-4	8.223	7.412	0.173	0.811
34	benzoic acid N,N-diethylamide (rebemide)	1696-17-9	6.659	6.495	0.517	0.165
35	3,6-dichloro-2-methoxybenzoic acid (dicamba)	1918-00-9	7.082	7.508	0.263	–0.426
36	1,4-benzenedicarboxylic acid (terephthalic acid)	100-21-0	8.071	7.469	0.153	0.602
37	2-hydroxy-5-methylbenzoic acid (p-cresotic acid)	89-56-5	6.908	7.516	0.156	–0.609
38	4-hydroxybenzoic acid propyl ester (propylparaben)	94-13-3	8.753	7.062	0.405	1.692
39	2-hydroxy-3-methylbenzoic acid (hydroxytoluic acid)	83-40-9	6.908	7.518	0.155	–0.610

**Table 3.** Toxicity prediction of the regression model with Testing dataset (18 benzoic acid compounds).

			**LogLD_50_**	
**No.**	**Compound**	**CAS No.**	**Dependent variable**	**Predicted value**
1	benzoic acid	65-85-0	7.57	7.16
2	2-benzoylbenzoic acid	85-52-9	6.68	5.69
3	2,3,5-triiodobenzoic acid	88-82-4	6.55	7.49
4	2-benzoyl-5-chlorobenzoic acid	1147-42-8	6.35	6.16
5	5-amino-2-benzoylbenzoic acid	2162-57-4	7.44	6.14
6	2-acetoxy-5-bromobenzoic acid	1503-53-3	6.48	7.43
7	4-methylbenzoic acid methyl ester	99-75-2	8.24	7.47
8	2-hydroxy-3,6-dichlorobenzoic acid	3401-80-7	6.49	7.40
9	benzoic acid 3-hydroxyphenyl ester	136-36-7	6.68	6.06
10	6-benzoyl-3-methylbenzoic acid	1147-41-7	6.80	5.28
11	3,4,5-trihydroxybenzoic acid propyl ester	121-79-9	7.44	6.78
12	2-hydroxybenzoic acid 2-methylpropyl ester	87-19-4	8.54	6.33
13	2-(3-chloro-2-methylphenylamino) benzoic acid	13710-19-5	5.63	4.52
14	3-acetylamino-2,4,6-triiodobenzoic acid (acetrizoate)	85-36-9	9.90	7.13
15	benzoic acid 2-methylpropyl ester (isobutyl benzoate)	120-50-3	8.48	6.50
16	2-(2,3-dimethylphenyl)aminobenzoic acid (mefenafic acid)	61-68-7	6.26	3.29
17	2-acetyloxy-4-trifluoromethylbenzoic acid (triflusal)	322-79-2	6.08	6.32
18	1,1′-biphenyl-2′,4′-difluoro-4-hydroxy-3-carboxylic acid (diflunisal)	22494-42-4	6.08	5.87
